# Clinical manifestation, staging and prognosis of hepatocellular carcinoma in Gambian patients

**DOI:** 10.1186/s12876-023-02952-8

**Published:** 2023-09-20

**Authors:** Sheikh Omar Bittaye, Abubacarr Kambi, Momodou A. I. Tekanyi, Saydiba Tamba, Lamin Sanneh, Momodou Musa Sisawo, Abdoulie Jatta, Gibril Fatty, Adam Jeng, Momodou Salieu Jallow, Ousman Leigh, Ramou Njie

**Affiliations:** 1 Department of Internal Medicine, Edward Francis Small Teaching Hospital, Banjul, The Gambia; 2 School of Medicine and Allied Health Sciences, University of The Gambia, Banjul, The Gambia; 3 Disease Control & Elimination, MRC Unit The Gambia @ London School of Hygiene & Tropical Medicine, Fajara, The Gambia; 4 Pathology Department, Edward Francis Small Teaching Hospital, Banjul, The Gambia; 5 American International University, Serekunda, Gambia

**Keywords:** Carcinoma, Hepatocellular, Hepatitis B, Survival

## Abstract

**Background:**

As a result of the lack of screening programs and the difficulty in making a proper diagnosis, the majority of hepatocellular carcinoma (HHC) patients present late in low-resource countries. The study therefore assesses the clinical features, stage and prognostic variables of patients with HCC in The Gambia.

**Methods:**

From December 2015 to January 2019, patients with a confirmed diagnosis of HCC were enrolled. All patients’ medical history, ultrasound scan, FibroScan and laboratory details were collected.

**Results:**

Two hundred and sixty (260) patients were enrolled. The mean age of HCC patients was 40 years, and 210 (80.7%) of them were male. The most common gastrointestinal symptoms were early satiety 229 (88.1%) and abdominal pain 288 (87.7%), while the most common constitutional symptoms were weight loss 237 (91.2%) and easy fatiguability 237 (91.2%). Hepatomegaly 205 (78.8%) was the most common sign. On ultrasound scan, lesions were mostly multifocal 175 (67.3%), and the median FibroScan score was 75 kPa. The median fibrosis 4 and aspartate transferase platelet ratio index were 4.6 and 2.2, respectively. Hepatitis B surface antigen (HBsAg) was positive in 170 (65.4%) patients, and the median AFP level was 3263 ng/ml. HCC patients with positive HBsAg were more likely to be male 145 (85.3%) vs 62 (72.1%) (*p* = 0.011), much younger 39.9 vs 51.4 yrs (*p* =  < 0.0001), more likely to have abdominal pain 156 (91.8%) vs 68 (79.1%) (*p* = 0.002), jaundice 78 (45.9%) vs 29 (33.7%) (*p* = 0.042), dark urine 117 (68.8%) vs 46 (53.5%) (*p* = 0.018), raised transaminases (Aspartate transaminases 224.5 (32–7886) vs 153 (18–610), *p* =  < 0.01, Alanine transferases 71 (5–937) vs 47 (8–271), *p* =  < 0.001) and decreased platelet count 207 (33–941) vs 252 (52- 641) (*p* = 0.021) compared to patients with HCC who were HBsAg-negative.

**Conclusions:**

The prognosis of patients with HCC is poor in developing countries such as The Gambia, where screening programs and treatment modalities are scarce. Young males are disproportionately affected, and HBV is a major cause of HCC in The Gambia.

## Background

Liver cancer was the fifth most common cancer in men and the seventh most common cancer in women worldwide in 2020 [1]. Most of the disease burden is in developing countries, where almost 85% of cases occur [2]. Hepatocellular carcinoma (HCC) represents over 80% of primary liver cancer cases [1, 3, 4] and is the third leading cause of cancer deaths worldwide [1], with a prevalence 16 to 32 times higher in developing countries than in developed countries [5]. Sub-Saharan Africa is one of the most affected region owing to the high prevalence of chronic hepatitis B virus infection [4, 6, 7].

Worldwide, chronic hepatitis B virus (HBV) infection accounts for approximately 50% of all cases of HCC [4, 8]. According to global estimates in 2015, the prevalence rate of hepatitis B in The Gambia was 12.28% [9]. A recently performed randomized controlled trial between 2011 and 2014 put the prevalence rate at 8.8% in the community and 12.6% among blood donors [10].

In countries that have adopted screening programs for liver cancer, there has been considerable change in the clinical manifestation over the past decade. Liver cancer patients diagnosed in non-screening programs have more symptoms/signs, more deranged liver function tests and worse survival than those diagnosed in screening programs [11].

Between 1990 and 2009, liver cancer was the most common cancer in men in The Gambia, accounting for approximately 60% of all cancer cases in men and approximately 24% of all cancer cases in women, following cervical cancer [8, 9]. HCC and chronic liver disease (CLD) account for 10 to 15% of all mortality in adult males [10].However, little is known about the clinical manifestations of patients with liver cancer in The Gambia.

Clinical staging of cancers provides a guide to assess prognosis and to direct therapeutic interventions. Several prognostic staging systems have been proposed for HCC and have been validated to be useful in prognostic stratification in different cohorts of patients [11] but have not been validated in The Gambia.

In developing countries, due to the scarcity or non-availability of surveillance/screening programs together with diagnostic challenges of liver cancer [12], most patients with liver cancer present late, which has an effect on the clinical manifestations, laboratory parameters, staging and prognosis of HCC patients. The study therefore assesses the clinical manifestations, staging and prognosis of HCC patients in The Gambia.

## Methods

### Patients

The study was a prospective cohort study conducted at the liver clinic of the Medical Research Council (MRC), Fajara, The Gambia, from December 2015 to January 2019. Patients diagnosed with HCC who were referred to the main liver clinic were recruited. The MRC is a research institution that has wards and an outpatient department. The main liver clinic was set up in 2012 by the International Agency for Research on Cancer (IARC) under the Gambia Hepatitis Intervention Study (GHIS) platform. This was the only specialist hepatology clinic that received referrals from across the whole Gambia during the study period.

### Diagnosis and staging of HCC

All HCC confirmed patients referred to the main liver clinic in MRC were recruited into the study after consenting. None of the patients declined. The diagnostic criteria of HCC were based on imaging techniques, including ultrasound scan, computerized tomography (CT), alpha-fetoprotein (AFP) and/or histology. The diagnostic criteria for HCC in this instance were ultrasound demonstration of a liver mass ≥ 2 cm with or without clinical features combined with an AFP level of ≥ 200 ng/ml and/or histopathological confirmation. Using a structured questionnaire, patients had their demographic information, aetiology of liver disease, clinical manifestations, FibroScan, imaging (ultrasound scan and/or CT scan), laboratory parameters (liver function test (LFT), full blood count (FBC) and AFP recorded at the time of presentation. Tumor characteristics on ultrasound scan were assessed by a hepatologist and trainee hepatologist. Their size, number and multifocal/unifocal pattern were recorded. All data, including staging of the tumour, were determined at the time of diagnosis. The symptomatic treatments received by patients were recorded. None of the patients had previous specific hepatitis B, hepatitis C or hepatocellular carcinoma treatment at the time of presentation or after diagnosis. Patients were also followed up to death, and the dates of death were documented. Patients were given appointments for follow-up at the clinic or field worker visits to the patient or by phone call.

### Laboratory investigations

Blood samples were collected from all patients, and LFT, FBC and AFP requests were filled and sent to the different labs (Haematology, Biochemistry and Serology). The finger-prick whole blood test for HBsAg was performed using a point-of-care test (Determine, Alere, Waltham, MA, USA), the performance of which has been validated in the field (sensitivity 88.5, specificity 100%) [10].AFP was detected and quantified by the ARCHITECT AFP assay, which is a chemiluminescent microparticle immunoassay (CMIA). Liver biopsy was only performed in patients with inconclusive diagnosis after signed informed consent was obtained. Liver biopsy samples were sent to Edward Francis Teaching Hospital for paraffin wax embedding. The paraffin wax blocks are then sent to IARC for hematoxylin & eosin staining and immunohistochemistry staining and reporting.

### Statistical analysis

Statistical analysis was performed using STATA/SE 12.1 statistics/data analysis. The Mann‒Whitney U test and Student’s t test were used for continuous variables with skewed and normal distributions, respectively. The chi-square test was used for discrete variables. Survival curves were drawn using the Kaplan–Meier method. In all cases, survival analysis was calculated from the date of diagnosis to the date of death or date of most recent follow-up for those lost to follow-up. Statistical significance was defined as *p* < 0.05.

## Results

### Demographic features of the study population

The study recruited 260 HCC patients, as shown in Table 1. Of these, 240 (92.3%) were diagnosed by ultrasound demonstration of a liver mass ≥ 2 cm with or without clinical features combined with an AFP level of ≥ 200 ng/ml, and the remaining 20 (7.7%) were diagnosed by ultrasound demonstration of a liver mass ≥ 2 cm with or without clinical features combined with histological confirmation. None of the patients had a triple phase CT scan.HCC patients were mostly male 210 (80.7%) and rural born 195 (75%). The majority were farmers/gardeners 98 (42%) and of the Wollof 68 (26.2%) or Mandinka 65 [13] tribes. The overall median age for HCC patients at the time of diagnosis was 40 years and ranged between 12 and 107 years. The median age for males was 40 years and that of females was 41 years, with a male to female ratio of 4:1.

**Table 1:** Demographic and clinical features of hepatocellular carcinoma patients

Variable	*n* = 260 (%)
**Age, yrs(range)**	40 (12–107)
**Age Group**	
< 35	73 (28.1)
35–44	84 (32.3)
45–54	47 (18.1)
55–64	25 (9.6)
≥ 65	31 (11.9)
**Sex**	
Male	210 (80.7)
Female	50 (19.3)
**Place of Birth**	
Rural	195 (75)
Urban	41 (15.8)
Foreign	24 (9.3)
**Ethnicity**	
Mandinka	65 (25)
Fula	55 (21.2)
Wollof	68 (26.2)
Jola	23 (8.9)
Others	49 (18.8)
**Marital status**	
Married	203 (79.9)
Single	34 (13.4)
Widow	11 (4.3)
Divorced	6 (2.4)
Missing	6
**Occupation**	
Farmer/Gardener	98 (42)
Construction/industrial	45 (19.3)
Business/Trader	27 (11.6)
Civil servant	24 (10.3)
Others	39 (16.7)
Missing	27
**Clinical features**	
**1. Constitutional symptoms**	
Fever	216 (83.1)
Night sweat	173 (67)
Weight loss	237 (91.2)
Easy fatiguability	237 (91.2)
**2. GI symptoms**	
Abdominal pain	228(87.7)
Early satiety	229 (88.1)
Abdominal distension	209 (80.3)
Anorexia	204 (78.5)
Nausea	161 (61.9)
Vomiting	97(37.3)
Bloody stool	74 (28.5)
Diarrhoea	89 (34.2)
Yellow eyes	110 (42.3)
Dark urine	166(63.8)
Bilateral leg swelling	93 (35.7)
Pruritus	79 (30.4)
**Examination**	
Oedema	89(34.2)
Jaundice	76(29.2)
Hepatomegaly	205(78.8)
Splenomegaly	28(10.7)
Ascites	89(34.2)
Collateral veins	43(16.5)
Abdominal tenderness	107(41.2)

Data are median (Range) or n (%)

### Clinical features

The most common constitutional symptoms were weight loss 237 (91.2%), easy fatiguability 237 (91.2%) and the most common gastrointestinal symptoms were early satiety 229 (88.1%) and abdominal pain 228 (87.7%). Abdominal distension 209 (80.3%) and anorexia 204 (78.5%) were also common. The most common signs were also hepatomegaly 205 (78.8%) and abdominal tenderness 107 (41.2%) (Table 1).

### Aetiology

Two hundred and fifty-six patients had a hepatitis B surface antigen (HBsAg) test. One hundred and seventy patients (65.4%) were positive for HBsAg. The remaining 86 patients were HBsAg negative (Table 2). Only 16 (6.1%) had a positive history of alcohol use. Of these, 5 (1.9%) had both a positive history of alcohol and a positive HBsAg.

**Table 2:** Ultrasound scan, non-invasive fibrosis tests and laboratory investigations

Variable	*n* = 260 (%)
**Ultrasound scan**	
Multiple lesions/Single lesion/No lesion	175 (67.3)/ 60 (23) / 25 (9.6)
**Fibroscan (*n* = 169)**	
Fibroscan (kPa): median (range)	75 (4–75)
**Fibrosis 4 (*n* = 231)**	
median (range)	4.6 (0.37–69.9)
> 1.45	202 (87.5)
> 3.25	143 (61.9)
**Aspartate transferase to platelet ratio (*n* = 232)**	
Median (range)	2.2 (0.1–102.7)
≥ 1.5	152 (65.5)
> 2	121 (52.2)
**Laboratory investigations**	
Hepatitis B surface antigen	
• Positive	170 (65.4)
• Missing	4(1.5)
Human immunodeficiency virus	
• Positive	11(5.2)
Alpha-fetoprotein (ng/ml)	3263 (1.07–116,000)
International normalized ratio	1.3(0.9–4.9)
Haemoglobin (g/dl)	11.3 (4.8–18.4)
White Cell Count (× 10^9^/l)	7.8 (1.8–34.8)
Red cell count (× 10^12^/l)	4.1 (0.9–7.7)
Platelet count(× 10^9^/l)	222(33–941)
Sodium(mmol/l)	139(121–151)
Potassium (mmol/l)	4.3 (3.36–10.6)
Creatinine (umol/l)	67(13–949)
Glucose (mmol/l)	4.5(0.9–13.4)
Aspartate Transferase (U/L)	194(18–7886)
Alanine Transferase ( U/L)	61(5–937)
Total Bilirubin (umol/l)	32(0–646)
Gamma glutamyl Transferase ( U/L)	315(11–3677)
Alkaline phosphatase (U/L)	282(28–4063)
Albumin (g/L)	33(10–52)

Data are median (Range) or n (%)

### Demographic, clinical and laboratory features of HCC patients with positive HBsAg compared to HCC patients with negative HBsAg

The median age of HCC patients who were HBsAg positive at diagnosis was 38 years compared to 50 years in those who were HBsAg negative. HCC patients with positive HBsAg decreased with increasing age. The male to female ratio was much more striking in HCC patients with positive HBsAg (6:1) than in HCC patients with negative HBsAg (3:1). HCC patients with positive HBsAg were mostly male 145 (85.3) vs 62 (72.1) (*p* = 0.011), much younger 39.9 vs 51.4 yrs (*p* =  < 0.0001), more likely to have abdominal pain 156 (91.8) vs 68 (79.1%) (*p* = 0.02), yellow eyes 78 (45.9%) vs 29 (33.7%) (*p* = 0.042), dark urine 117 (68.8%) vs 46 (53.5%) (*p* = 0.018), abdominal tenderness 83 (48.2%) vs 22(25.8%) (*p* = 0.001), increased aspartate transferase (AST) 224.5 (32–7886) vs 153 (18–610) (*p* =  < 0.001), increased alanine transferase (ALT) 71 (5–937) vs 47 (8–271) (*p* =  < 0.001) and decreased platelet counts 207 (33–941) vs 252(52- 641)(*p* = 0.021) compared to HCC-negative HBV patients (Table 3).

**Table 3:** Demographic, clinical and laboratory features of HCC patients with positive HBsAg as compared to HCC patients with negative HBsAg

	HCC with positive HBsAg (*n* = 170)	HCC with negative HBsAg (*n* = 86)	*P* value
**Age(yrs)Mean**	39.9	51.4	< 0.0001
**Age groups**			
< 35	59(34.7)	13(15.1)	< 0.0001
35–44	65(38.2)	18(20.9)	
45–54	28(16.5)	19(22.1)	
55–64	12(7.1)	11(12.8)	
≥ 65	6(3.5)	25(29.1)	
**Sex (M:F)**	145(85.3):25(14.7)	62(72.1):24(27.9)	0.011
**Clinical features**			
**1. Constitutional symptoms**			
Fever	143(84.1)	69(79.1)	0.480
Night sweat	116(68.2)	54(62.8)	0.414
Weight loss	154(90.5)	79(91.9)	0.723
Easy fatiguability	153(90)	80(93)	0.328
**2. GI symptoms**			
Abdominal pain	156(91.8)	68(79.1)	0.002
Early satiety	150(88.2)	75(87.2)	0.801
Abdominal distension	139(81.8)	66(76.7)	0.329
Anorexia	137(80.6)	63(73.3)	0.170
Nausea	108(63.5)	51(59.3)	0.468
Vomiting	65(38.2)	32(37.2)	0.955
Bloody stool	49(28.8)	24(27.9)	0.922
Diarrhoea	64(37.6)	24(27.9)	0.112
Yellow eyes	78(45.9)	29(33.7)	0.042
Dark urine	117(68.8)	46(53.5)	0.018
Pruritus	50(29.4)	27(31.4)	0.677
Oedema	58(34.1)	34(39.5)	0.392
**Medical history**			
Hypertension	8(4.7)	10(11.6)	0.039
Anaemia	44(25.9)	20(23.3)	0.664
Cigarette smoking	68 (40)	27 (31.4)	0.215
Alcohol	11(6.4)	5(5.8)	0.865
**Examination**			
Oedema	53 (31.2)	35( 40.7)	0.100
Jaundice	54 (31.8)	21(24.4)	0.239
Hepatomegaly	132 (77.6)	70 (81.4)	0.236
Splenomegaly	19 (11.2)	9 (10.5)	0.887
Ascites	58 (34.1)	29(33.7)	0.973
Collateral veins	30 (17.6)	13 (15.1)	0.620
Abdominal tenderness	82 (48.2)	22(25.6)	0.001
**Laboratory investigations**			
Alpha-fetoprotein (ng/ml)	4780.48 (3- 116000)	2527.46(1.07–94600)	0.385
Haemoglobin (g/dl)	11.4( 4.8–18.4)	11.2 ( 5.6–16.8)	0.248
White Cell Count (× 10^9^/l)	8.25( 1.8–34.8)	7.6 (2.2- 27.8)	0.238
Red cell count (× 10^12^/l)	4.21(0.9- 7.67)	4.01(2.34–6.9)	0.314
Platelet count (× 10^9^/l)	207(33–941)	252(52- 641)	0.021
Sodium (mmol/l)	138(122–151)	139(121–147)	0.228
Potassium (mmol/l)	4.4(2.36–10.6)	4.1(3.1–6.1)	0.074
Creatinine( umol/l)	65( 13–949)	68.5(37–744)	0.247
Glucose (mmol/l)	4.45( 0.9–13.4)	4.65(2.7–8.4)	0.105
Aspartate Transferase (U/L)	224.5(32–7886)	153(18–610)	< 0.001
Alanine Transferase (U/L)	71(5–937)	47(8–271)	< 0.001
Total Bilirubin (umol/l)	37.5(0–646)	25(6–273)	0.0625
Gamma glutamyl Transferase (U/L)	305(14–1826)	344(11–3677)	0.851
Alkaline phosphatase (U/L)	281(28–4063)	282(70–1902)	0.820
Albumin (g/L)	33(15–52)	33(20–45)	0.551
**Survival**			
Median survival (Days)	31(1–448)	41(3–510)	0.175

Data are median (Range) or n (%)

### Laboratory investigations

The laboratory investigations of all patients are summarized in Table 2. The median AFP was 3263 ng/ml (1.07–116,000). The median AST and ALT levels were 194 U/L (18–7886) and 61 U/L (5–937), respectively. The AST/ALT ratio was 3.2:1. The median gamma-glutamyltransferase (GGT) level was 315 U/L (11–3677), and the median alkaline phosphatase (ALP) level was 282 U/L (28–4063). However, median albumin was low (33 g/dl). Among the two hundred and eleven patients who had HIV tests, 11 patients were positive (5.2%).

### Ultrasound scan and non-invasive fibrosis tests

Multifocal lesions 175 (67.2%) were the most common on ultrasound scans compared to uni- focal lesions 60 (23%). In 25 (9.6%) of the patients, no obvious lesion was seen on ultrasound scan. The median FibroScan score in our patients was 75 kPa (4–75). The median fibrosis 4 score (FIB 4) was 4.6 (0.37–69.9), and the majority 143 (61.9%) of the patients had a score greater than 3.25. The median aspartate transferase to platelet ratio index (APRI score) was also 2.2 (0.1–102.7), with most 121 (52.2%) patients having a score greater than 2 (Table 2).

### Clinical staging and survival analysis

**Fig. 1:**
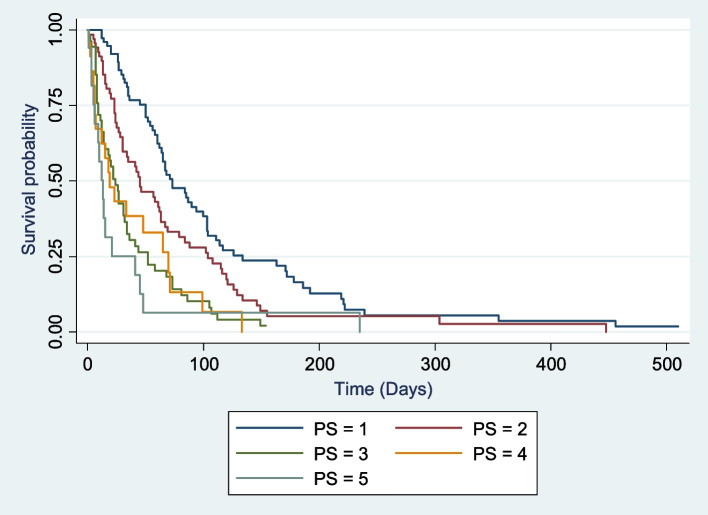
Survival of HCC patients according to WHO performance status (PS)

**Fig. 2:**
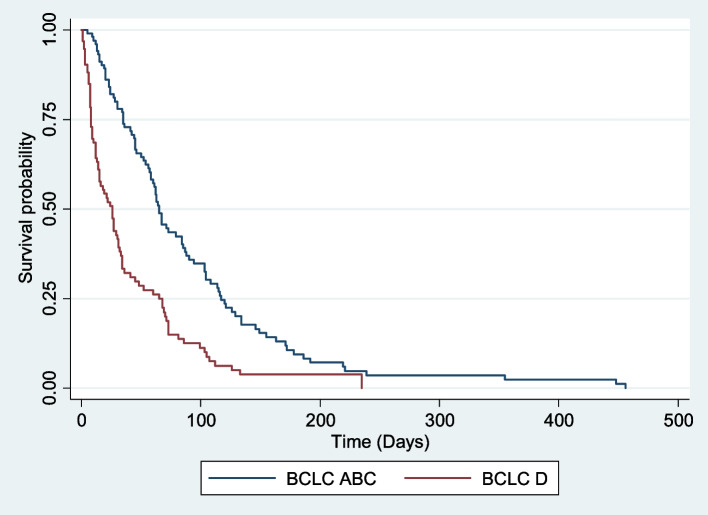
Survival of HCC patients according to Barcelona Clinic Liver Cancer stage (BCLC)

**Fig. 3:**
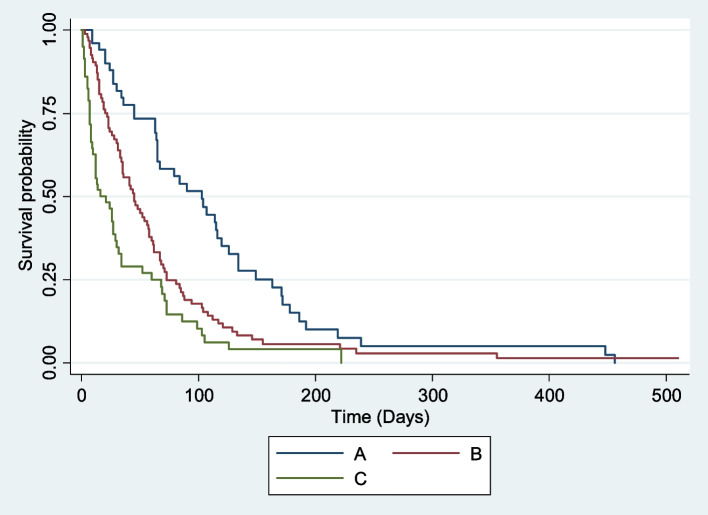
Survival of HCC patients according to child–pugh class

The most common World Health Organization (WHO) performance status was 2 69 (28%), and most of the patients had a WHO performance status > 1 165 (67.1%). The majority 191 (94%) of the patients had Barcelona Clinic Liver Cancer (BCLC) stage C 94 (46.2%) or BCLC stage D 97 (47.8%). Child‒Pugh stage B 97 (46.4%) was also the most common, with a median Child‒Pugh score of 8 (Table 4). Both prognostic stages and WHO performance status had good stratification for survival (Figs. 1, 2 and 3). None of the patients met the Milan criteria at presentation. The overall median survival of these patients was 33 days (Table 4). Only 39 patients (15%) of the 260 patients were lost to follow-up, and the median duration from presentation to lost to follow up was 21 days. The median survival of the 221 (85%) patients whose dates of death were known was 35 days.

**Table 4:** Clinical staging and survival analysis

Variable	n (%)
**Clinical staging**	*n* = 246
Performance status (%): 0/1/2/3/4/5	18 (7.3)/63 (25.2)/69 (28)/54 (22.4)/25 (10.2)/17 (6.9)
	*n* = 203
Barcelona clinic liver cancer staging (%): A/B/C/D	4 (1.9)/8 (3.9) /94 (46.3)/97 (41.8)
	*n* = 209
Child pugh Scoring (%): A/B/C	52 (24.9)/ 97 (46.4) /60 (28.7)
Child Pugh score	8 (5–13)
**Survival (Days)**	*n* = 260
Overall median survival	33 (1–510)

Data are median (Range) or n (%)

## Discussion

This study presents the data of 260 HCC patients, which strongly showed that hepatitis B virus is an important aetiological factor for HCC patients in The Gambia and that young males are disproportionately affected. The study also revealed that the prognosis of HCC patients in The Gambia is very poor and that most of them present late with clinical, laboratory and radiological features consistent with multiple/large tumours and hepatic decompensation at the time of presentation.

The demographics of our patients were consistent with previous studies [14, 15]. Males were more affected and were much younger at presentation than females. The difference in sex is not well understood, but it has been postulated that 1) the decrease in adiponectin levels in males may be responsible for the increase in liver cancer risk. The decrease in adiponectin is due to the testosterone activation of JNK, mediating the inhibition of adiponectin secretion, which increases liver cell proliferation [16]. 2) Other studies have suggested that oestrogen acts as an inhibitor of the proliferation, growth and metastasis of HCC cells and can prevent liver cancer development [17]. The median age in our cohort was younger, and there was also a difference in ethnicity compared to a previous study, the Gambia liver cancer study (GLCS) 1997–2001 [12]. The difference in age may be because approximately 80% (32% between 35–44 years) of our patients were < 54 years compared to 65% < 54 years (25% between 45–54 years).The age differences may also reflect the fact that in the GLCS, a significant (19%) of HCC causes were attributed to HCV, which affected mainly older patients, although this was not routinely tested in this cohort [15]. The fact that many more patients from the original GHIS cohort are now presenting with liver disease, which is being captured, may also be a contributory factor to the lower age group in this cohort. The difference in ethnicity could also be explained by the inclusion of Farafenni Hospital as one of our recruitment sites where we had a sizeable number of Wollof and Mandinka.

The most common symptoms were weight loss, easy fatiguability, abdominal pain, abdominal distension and early satiety, while the most common signs were also hepatomegaly and abdominal tenderness. This suggests that the majority of our patients had clinical manifestations related to the tumour at the time of presentation. These findings are consistent with the clinical presentation of patients in resource-limited countries such as The Gambia, where screening programs and therapeutic interventions are limited [11]. In resource-rich countries, with the advent of surveillance with ultrasound scans and AFP in patients with chronic hepatitis, the clinical manifestation of patients has changed significantly. The patients in the screening groups are found to have fewer symptoms and signs, fewer deranged biochemical indices, a better chance of receiving HCC treatment and better survival [11].

The HBsAg carriage in this study was 65.4%, which is similar to previous studies [15, 18]. In another study, HBsAg carriage was lower 50% [19], which could be due to the small sample size. Studies performed in Senegal, Ghana and Nigeria reported hepatitis B positivity rates of 69.5%, 75.2% and 52%, respectively, in HCC patients [20–22]. This confirms hepatitis B as a major risk factor for HCC in The Gambia and the subregion. In this study, only 6.1% had a positive history of alcohol use. This is different from Ghana, where 39.2% admitted to taking alcohol. Compared to hepatitis B, alcohol seems to play a minor role in the development of HCC in The Gambia. However, in 2019, alcohol was associated with approximately one-fifth of global HCC-related deaths [23]. The study did not examine the role of HCV and aflatoxin, and thus, there is a need for further studies to investigate their role.

HBsAg-positive patients with HCC were mostly males, much younger, most likely to have abdominal pain, jaundice, dark urine and abdominal tenderness compared to HBsAg-negative patients with HCC. Patients diagnosed with HCC who were HBsAg-negative tended to be older, more likely to be hypertensive and had a much better median survival (41 days vs 31 days). The clinical picture of HBsAg-positive patients with HCC is more aggressive than that of HCC patients with negative HBsAg. This further justifies the need to establish screening programmes for those with chronic hepatitis B infection [10, 11, 24]. The male to female ratio and median age at presentation of HBsAg-positive patients with HCC in this study were similar to those in a previous study [15]. The male to female ratio was also much more striking in HCC patients with positive HBsAg (6:1) than in HCC patients with negative HBsAg (3:1).Males are more likely to experience chronic hepatitis B carriage [10, 25]. However, further research needs to be done to investigate the higher incidence of chronic liver disease and HCC in males.

The percentage of HCC patients who are HBsAg positive has fallen to approximately 80% in the < 45-year-old age group compared to > 90% in the GCLS study. There was, however, a significant increase in the percentage of HBsAg-positive HCC patients in the 55–64 year age group, from 20% in the GCLS to 50% in the current study [13, 15]. The reason for this could be explained by the hepatitis B vaccination effect, which started in 1986 [25–28], and the aging of the non-vaccinated cohort.

The laboratory investigations also showed higher transaminase and cholestatic enzymes and hypoalbuminemia, which is consistent with hepatic decompensation in our patients at the time of presentation. The AST/ALT ratio was also high. As already known, high GGT and AST/ALT are found to be independent factors for predicting poor overall survival of primary hepatic carcinoma patients [29].HBsAg-positive patients with HCC were also more likely to have increase transaminases and decreased platelet count as compared to HBsAg-negative patients with HCC. Decreased platelet count is link to poor survival and is a common finding in patients with advanced fibrosis and portal hypertension [30].These findings confirms the fact that most of our patients, especially those with positive HBsAg presents with hepatic decompensation and thus has a very poor overall survival.

Most of the patients also had multifocal lesions with a median FibroScan score of 75 kPa. A majority 143 (61.9%) of the patients had an FIB 4 score greater than 3.25. One hundred and twenty-one (52.2%) patients also had APRI scores greater than 2. The above findings suggest that most of our patients developed significant fibrosis/cirrhosis at the time of presentation. As already known, a high FIB 4 score [31] or APRI score [32, 33] has been associated with poor survival. This further confirms the fact that most HCC patients present late and thus have a poor prognosis at the time of presentation.

The overall median survival of our patients was 33 days. This is very poor when compared to other studies [11, 34–37] but was similar to HCC patients who received supportive treatment only in Ethiopia [38]. Even among African countries, the overall median survival of HCC patients in Egypt was significantly longer when compared with other African countries [37]. The reasons for this could be 1) the absence of a nationwide screening, treatment and surveillance programme for hepatitis B, which is the main aetiology for HCC in The Gambia; 2) the absence of skilled manpower and infrastructure, surgical and other therapeutic interventions such as transarterial chemoembolization, radiofrequency ablation therapy, liver transplantation and other surgical interventions and 3) the late presentation of our patients, which could be because they first consult a local herbalist before going to a health facility. However, there is a dire need for the discovery of new variables/biomarkers that can aid in early diagnosis and prognosis in countries without screening/surveillance.

WHO performance status > 1, advanced or terminal BCLC stages (C and D) and Child‒Pugh stage B were most common among our patients, and none of our patients met the Milan criteria. The findings are similar to studies done in Sub-Saharan Africa [20, 37] but are much different from most other studies done in the developed world [11, 34–36]. This suggests that the majority of our patients are diagnosed late, and thus, the only therapeutic option available for them is symptomatic treatment. WHO performance status, Child‒Pugh class and BCLC all had good stratification of survival across all stages of the disease. Upon further analysis of the different staging systems using Kaplan‒Meier survival analysis, each system showed a significant difference in survival across different stages. The median survival of the different stages in our cohort was worse than that seen in Hong Kong and Japan [11, 34, 36]. The WHO performance status, BCLC stage and Child‒Pugh stage were all worse in other African countries than in Egypt [37]. The above finding confirms that countries with higher socioeconomic status have better survival rates, and even in resource-limited countries, there exists a difference in the survival of HCC patients.

The study has some limitations: 1) The diagnostic criteria used in this study were based on a combination of clinical, ultrasound scan, AFP level and, in a few cases, histology and not the preferred triple phase CT scan, which was not available during the study period, 2) the study did not also include other aetiologies, such as hepatitis C and aflatoxin, which could have also contributed to some of the HCC cases and 3) It is also possible that patients with early stage HCC could have been missed on ultrasound scan due to several factors, such as its limitation in diagnosing early stage HCC and operator skill, which affect the sensitivity of ultrasound scan in the HCC diagnosis.. Notwithstanding, the study is most likely representative of the whole country since patients with suspected HCC from health facilities across the country were referred to the only specialist hepatology clinic during the study period. Second, the study gave us a detailed understanding of the clinical features and prognosis of HCC in The Gambia, which can be compared with other studies in the future.

In conclusion, chronic hepatitis B infection is still the major factor responsible for the development of HCC in The Gambia. HBsAg-positive patients with HCC were mostly young males who are more likely to be symptomatic and have a much shorter survival. Due to the absence of nationwide screening/surveillance and treatment programs for both hepatitis B and HCC, the survival and prognosis of HCC is poor in the Gambia. This study thus justifies the need to establish preventive, screening, treatment and surveillance programmes in resource-limited countries such as The Gambia.

## Data Availability

The dataset for this publication is available on reasonable request from the corresponding author.

## Abbreviations

MRC: Medical research council
GHIS: Gambia hepatitis intervention study
IARC: International Agency for Research on Cancer
HBV: Hepatitis B virus
HCC: Hepatocellular carcinoma
CLD: Chronic liver disease
GLCS: Gambia liver cancer study
AFP: Alpha-fetoprotein
CT: Computerized tomography
LFT: Liver function test
FBC: Full blood count
CMIA: Chemiluminescent microparticle immunoassay
HIV: Human immunodeficiency virus
WHO: World Health Organization
BCLC: Barcelona Clinic Liver Cancer Staging
HBsAg: Hepatitis B surface antigen
ALP: Alkaline phosphatase
GGT: Gamma glutamyltransferase
AST: Aspartate transferase
ALT: Alanine transferase
